# Aseptic Meningitis, Mucocutaneous Lesions and Arthritis after COVID-19 Vaccination in a 15-Year-Old Boy

**DOI:** 10.3390/vaccines10020325

**Published:** 2022-02-18

**Authors:** Thomas Bogs, Nadia Saleh, Suleyman Tolga Yavuz, Walid Fazeli, Rainer Ganschow, Felix Schreiner

**Affiliations:** 1 Department of Pediatrics, Children’s Hospital, University of Bonn, 53127 Bonn, Germany; thomas.bogs@ukbonn.de (T.B.); nadia.saleh@ukbonn.de (N.S.); s.tolga.yavuz@ukbonn.de (S.T.Y.); rainer.ganschow@ukbonn.de (R.G.); 2 Department of Neuropediatrics, Children’s Hospital, University of Bonn, 53127 Bonn, Germany; walid.fazeli@ukbonn.de

**Keywords:** aphthous ulcers, aseptic meningitis, Behçet’s disease, mucocutaneous lesions, COVID-19, vaccination, mucocutaneous lesions

## Abstract

We report a 15-year-old boy who developed aseptic meningitis 10 days after administration of the second dose of the COVID-19 vaccine BNT162b2. Although accompanying aphthous mouth ulcers resembling herpetic stomatitis initially led us to suspect an underlying viral infection, broad virological and microbiological screening did not identify any causative pathogen. Gonarthritis and skin lesions, which both developed within three days after admission, extended the clinical presentation eventually resembling an acute Behçet’s disease episode. This is the first description of a juvenile patient with aseptic and pathogen-negative meningitis occurring in close temporal association with vaccination against COVID-19, along with a few previously reported adult patients with isolated meningitis and a further case with meningitis and an accompanying Behçet’s disease-like multisystem inflammation episode as seen in our patient. With billions of individuals being vaccinated worldwide so far and only a few cases of aseptic pathogen-negative meningitis reported in close temporal relation, causality is unclear. However, aseptic meningitis should be kept in mind in the differential diagnosis of patients with persistent or delayed onset of headache and fever following COVID-19 vaccination.

## 1. Introduction

Vaccination against COVID-19 is essential to control the pandemic. The vaccines developed so far have good safety profiles, but full knowledge of adverse effects will only be acquired with time and through reports of unexpected medical events occurring in temporal association with their administration. Along with an increasing number of individuals being immunized worldwide, descriptions of rare but serious neuroinflammatory conditions such as Guillain–Barré syndrome (GBS) and transverse myelitis observed in close temporal relation with COVID-19 vaccinations led to considerations on specific neurological adverse reactions, although causality is still a matter of debate [1,2]. Recently, a few adult patients with aseptic meningitis possibly triggered by a COVID-19 vaccination have been described [3,4,5,6,7], one of which presented additional mucocutaneous and ocular symptoms leading the authors to consider the differential diagnosis of a first episode of Behçet’s disease [7]. Here, we report the first juvenile patient who developed aseptic meningitis with accompanying mucocutaneous lesions and arthritis resembling a Behçet’s disease episode in close temporal association with the COVID-19 vaccine BNT162b2 (Comirnaty, Pfizer/BioNTech, New York, NY, USA/Mainz, Germany).

## 2. Case Report

**Figure 1:**
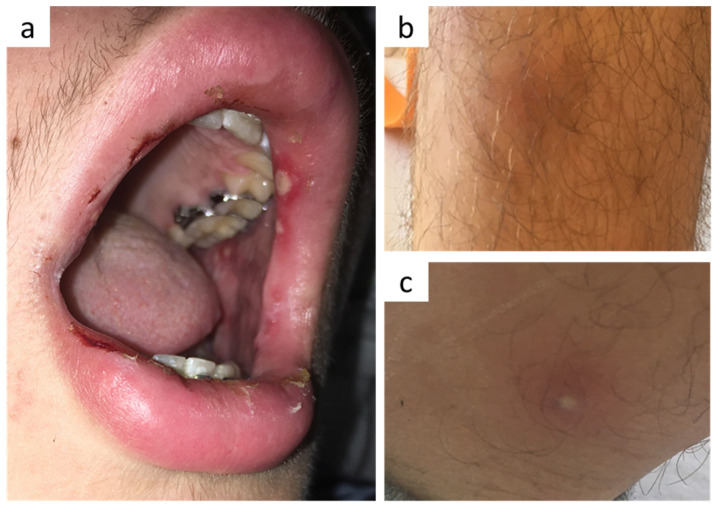
(**a**) Oral aphthous ulcers on lips and inner cheeks, (**b**) erythema nodosum-like lesions and (**c**) a single pustular lesion on his lower legs.

Thirteen days after receiving his second dose of the BNT162b2 vaccine in late October 2021, a 15-year-old boy of German descent presented at our hospital with acute headache for three days and fever, nausea, vomiting as well as oral aphthous ulcers (Figure 1a) for one day. Ibuprofen did not improve the symptoms. His further medical history was unremarkable except for a knee cartilage surgery approximately one month ago due to a sports accident and a few tick bites. Vaccinations were complete according to the recommendations of the German Standing Committee on Vaccination (STIKO), including chickenpox immunization.

Upon admission, neck rigidity, positive Kernig and Brudzinski signs together with nausea and fever of 38.8 °C led to suspicion of meningitis. Aphthous ulcers were detected on lips, tongue and inner cheeks (Figure 1a), but not in the palate area. Further physical examination including skin status and neurological functions was unremarkable at this time. Blood count showed a mild leukocytosis of 10,920/µL (ref. 4200–10,800/µL) with predominant neutrophils of 8980/µL, elevated C-reactive protein (5.34 mg/dL, ref. <0.3 mg/dL), and normal procalcitonin level (<0.5 µg/L). Cerebrospinal fluid (CSF) analysis revealed a pleocytosis of 242/µL white blood cells, consisting of both granulocytes (109/µL) and lymphomononuclear cells (133/µL). Whereas liquor lactate (2.92 mmol/L) and glucose (65 mg/dL) were largely within normal ranges, concentrations of protein (69.4 mg/dL, ref. 15–40 mg/dL) and CSF/serum indices for IgA and IgM were significantly increased.

Broad microbiological and virological screening did not identify any causative pathogen. Screening included negative bacterial cultures and 16S ribosomal DNA-PCR as well as virological PCR analyses for HSV-1/2, HHV-6/7, parvovirus B19, EBV, VZV, CMV, entero-, parecho-, adeno-, noro-, measles, mumps, FSME-, and west-nil-virus in appropriate specimens (CSF, blood, stool and/or aphthous ulcers). Similarly, serological analyses did not reveal evidence for acute infections with VZV, HSV, CMV, EBV and *B. burgdorferi* (the latter including negative chemokine CXCL13 in CSF and persistently negative IgM and IgG in a control serum after 3 weeks). PCR and serological tests for an acute and/or prior COVID-19-infection were also unremarkable. Finally, a next-generation sequencing-based pathogen detection kit (DISQVER^®^, Noscendo, Duisburg, Germany) that amplifies free DNA from microbiological organisms and DNA-viruses did not identify a causative infectious agent in a blood sample taken due to persistence of nocturnal fever peaks three days after initiation of antibiotic treatment with cefotaxime.

During the three days following admission, the patient developed a slight maculopapular exanthema on his cheeks (not shown), a few erythema nodosum-like lesions (Figure 1b) and a single pustular lesion (Figure 1c) on his lower legs as well as swelling of his right knee, which had been operated one month before. Ultrasound revealed echo-free joint effusion (aspiration was refused by the patient and his parents).

**Figure 2:**
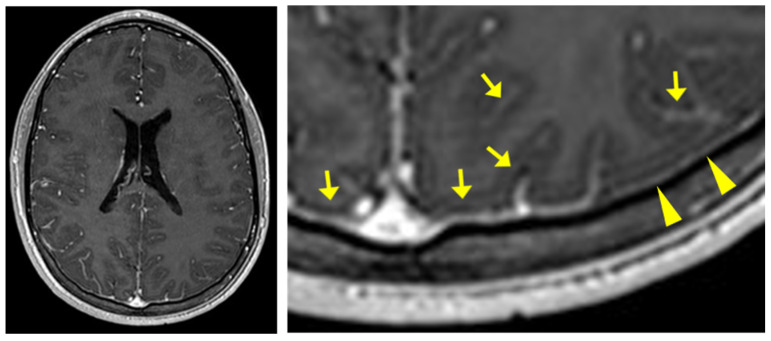
cMRI with a gadobutrol-enhanced T1-weighted fastfield echo (T1-FFE) sequence performed three days after lumbar puncture showing slight pachy- (arrowheads) and leptomeningeal (arrows) enhancement.

He also complained of vitreous floaters. Thorough ophthalmological examination did not reveal signs of uveitis or other intraocular abnormalities, except for minimal inhomogeneities in the posterior vitreous detected by optic coherence tomography (OCT). Cranial MRI showed slight pachy- and leptomeningeal enhancement but otherwise normal intracranial morphology (Figure 2). Autoimmune serological analyses for rheumatoid factor, ANA, pANCA, cANCA, and anti-dsDNA, as well as genotyping for HLA-B51, were negative.

From the third to the fifth day after admission, fever and headache, as well as the abovementioned mucocutaneous, visual and joint complaints markedly decreased. Antibiotic treatment with cefotaxime was terminated after a total of eight days, whereas acyclovir was given only two days until negative PCR analyses for HSV and VZV were obtained. The boy fully recovered within three weeks after admission and remained asymptomatic for the following two months.

## 3. Discussion

We report a 15-year-old Caucasian boy who developed aseptic meningitis, arthritis and mucocutaneous lesions, resembling an acute episode of Neuro-Behçet’s disease, in close temporal relation to the second dose of the COVID-19 vaccine BNT162b2. Although we are aware that the association of this inflammatory condition with COVID-19 vaccination may be coincidental, some aspects need to be discussed in more detail.

To date, all reported cases of aseptic and pathogen-negative meningitis occurred after immunization with mRNA vaccines [3,4,5,6,7], and all except one [7] after the BNT162b2 vaccine. This may simply reflect the fact that BNT162b2 currently is the most commonly administered COVID-19 vaccine in many regions worldwide. However, certain rare neurological conditions associated with COVID-19 vaccinations such as cerebral venous sinus thrombosis or Guillain–Barré syndrome (GBS) appear to be linked to specific vaccines or vaccine types [1,2]. Patone et al. also analyzed the frequencies of encephalitis-, meningitis- and myelitis-related admissions (*n* = 1285) that occurred in timely relation to first-dose vaccinations with any of the two aforementioned vaccines (*n* = 32,552,534, December 2020 to May 2021) as well as a COVID-19 infection itself. In the 1–28-day period after vaccination, they did not find an association with ChAdOx1-S (Vaxzevria, AstraZeneca, Cambridge, UK) or BNT162b2, but a significantly increased incidence rate after COVID-19 infection [2]. The Vaccine Adverse Event Reporting System, which is a passive national warning system in the US that records entries of healthcare professionals, vaccine manufacturers and the public, currently (18 December 2021) lists a total of 41 reported events with the symptom “aseptic meningitis” after a COVID-19 vaccination [8]. Of them, 16 events were associated with BNT162b2, 22 with mRNA-1273 (Spikevax, Moderna Biotech, Cambridge, MA, USA), and another three with the Ad26.COV2.S (Janssen, Johnson & Johnson, New Brunswick, NJ, USA) vaccine. A comparison with the relative distribution of administered vaccine doses in the US does not reveal an unequal risk distribution between the vaccines. However, the term “aseptic meningitis” is generally used to describe meningitis that is not caused by (pyogenic) bacteria, and in many cases a virus infection (e.g., enterovirus) can be detected. A modified VAERS database search for “viral menigitis” currently yields a total of 32 events. However, due to an unknown number of double entries, we do not know the exact number of aseptic virus-negative meningitis cases.

Table 1 displays clinical and biochemical characteristics of our case in comparison with the five previously reported patients with aseptic and pathogen-negative meningitis (Table 1). Saito et al. reported a considerable fraction of granular cells in the CSF pleocytosis [6], as also seen in our patient, whereas the remaining patients presented with an almost exclusive mononuclear cell pleocytosis [3,4,5,7]. On the other hand, the patient of Saito et al. is the only one who was reported to have a normal CSF protein level [6]. In addition, clinical meningitis symptoms in two patients [3,5] were already present on the first day after vaccine administration, as opposed to a period of at least one week in the other patients [4,6,7]. Of note, the patient reported by Tagini et al. did not present clinical signs of meningitis, and CSF analysis was primarily performed due to ophthalmological and cMRI findings suggestive of intracranial hypertension. Although the authors interpret the mild pleocytosis as aseptic meningitis [7], pleocytosis may accompany benign intracranial hypertension in some cases [9]. These findings, together with the presence of accompanying inflammatory manifestations, particularly of skin and mucosa in a subset of cases as discussed below, seem to indicate different underlying pathomechanisms of the meningeal inflammation between these patients.

After a few days without clinical improvement and with negative microbiological and virological analyses, the authors in three out of four previous cases with isolated aseptic meningitis speculated on a primary immune-reactive etiology and initiated immunosuppressive treatment with corticosteroids, which led to a rapid decrease in inflammation markers and clinical symptoms [3,5,6]. In our patient, meningitis was accompanied by impairment of other organs, including oral mucosa, skin, knee joint and vitreous body, resembling a Behçet’s disease episode. However, because some of these symptoms (maculopapular lesion, joint effusion, vitreous floaters) occurred only with delay and then spontaneously started to regress within two to three days after their onset (i.e., before all microbiological and virological analyses were completed), we did not consider immunosuppressive treatment for a possible multiorgan autoinflammatory condition. Based on the current clinical scoring system as proposed by the International Team for the Revision of the International Criteria for Behçet’s Disease (ITR-ICBD), our patient has an ICBD-score of 4 (2 points for oral aphthosis, 1 point each for skin lesions and neurological manifestation), which is exactly the cutoff value (≥4) to indicate a Behçet’s Disease diagnosis [10]. However, the predictive value of diagnostic criteria sets in regions where the disease is rare may be comparatively low and older diagnostic criteria such as those of the International Study Group of Behçet’s Disease (ISG), which required the presence of recurrent oral ulcerations plus any two of genital ulcerations, typical defined eye or skin lesions, or a positive pathergy test, would not have allowed the diagnosis in the present case, not least because of the lack of recurrent symptoms [11]. As already discussed by Tagini et al., the diagnosis of Behçet’s disease in a constellation of a first episode following vaccination, i.e., in the absence of recurrence, is rather unlikely. In their case description, Behçet’s disease was considered to be more likely due to the persistence of intracranial hypertension and the apparition of retinitis [7]. HLA-B51 genotyping was negative in both patients (ref. [7] and the current case), albeit generally one to two thirds of patients with Behçet’s Disease are HLA-B51 negative and the HLA-B51 status also does not seem to correlate with neurological involvement [12].

Particularly oral side effects including aphthous ulcers, blisters and plaques appear to be not uncommon after COVID-19 vaccinations. A cross-sectional survey of 877 healthcare professionals in the Czech Republic revealed a total of 114 persons who reported oral side effects during a period of four weeks after administration of the BNT162b2 vaccine, of which blisters (36%), ulcers (14%) and plaques (10.5%) were among the most common findings [13]. On the other hand, there are to date only a few case reports of isolated vulvar aphthous ulcer after administration of the BNT162b2 vaccine in otherwise healthy adolescents [14,15,16], one of which had a history of recurrent oral ulcerations. Watad et al. reported four patients from Israel with flares of Behçet’s disease (mainly oral aphthous ulcers of moderate severity), along with a large case series of flares or manifestations of other immune-mediated diseases following COVID-19 vaccination, and discussed a potential role of the Toll-Like Receptor (TLR) 7 and 9 adjuvanticity of the modern COVD-19 vaccines [17]. It will be interesting to follow similar future surveys of mucocutaneous lesions and Behçet’s disease-like episodes after COVID-19 vaccinations in regions with even higher prevalence of Behçet’s disease such as Turkey. Longitudinal observations of rare and apparently single episodes, as presented by our patient, are needed to clarify the individual risk of developing chronic autoinflammatory disease.

## 4. Conclusions

Here, we report a juvenile patient who developed aseptic meningitis with accompanying mucocutaneous lesions and arthritis resembling a Behçet’s disease episode in temporal association with the BNT162b2 vaccine. Although causality is unclear, aseptic meningitis should be kept in mind in the differential diagnosis of patients with persistent or delayed onset of headache and fever following COVID-19 vaccinations. The multisystem inflammation resembling Behçet’s disease, as presented by two out of six reported patients so far, warrants a more focused survey of this condition in regions with high prevalence of Behçet’s disease.

## Figures and Tables

**Table 1:** Reported patients with aseptic meningitis after COVID-19-vaccinations.

	Saito et al.	Lee	Reis Carneiro et al.	Tagini et al.	Dupon et al.	Current Case
Age/sex	42 y/female	18 y/male	62 y/female	late 20s/female	34 y/female	15 y/male
Vaccine	BNT162b2	BNT162b2	BNT162b2	mRNA-1273	BNT162b2	BNT162b2
Onset of symptoms ^#^	7 days	3 weeks	1 day	1–2 weeks	6–8 h	10 days
Vaccine dose	1st	2nd	1st	2nd	2nd	2nd
Preexisting conditions	migraine	-	dyslipidemia, anxiety	polycystic ovary syndrome	-	knee surgery 1 month ago
Accompanying Symptoms	-	-	-	oral and genital aphthous ulcers, pseudofolliculitis, intracranial hypertension	joint pain in wrists, ankles and knees, petechiae on lower extremities	oral aphthous ulcers, pustular skin lesion, erythema nodosum-like lesions, joint effusion, vitreous floaters
C-reactive protein	9.85 mg/dL	0.74 mg/dL	n.r.	10.9 mg/dL	16.9 mg/dL	5.34 mg/dL
CSF pleocytosis	176/µL (64.1% mononuclear, 34.8% granular)	115/µL (99.1% mononuclear)	1st LP: 101/µL 2nd LP: 301/µL (100% lymphocytes)	27/µL (98% lymphocytes, 2% monocytes)	188/µL (lymphocytic pattern)	242/µL (55% mononuclear, 45% granular)
CSF protein level	35.7 mg/dL (ref. 0–45)	67.2 mg/dL (ref. 20–45)	1st LP: 154 (ref. 15–40) 2nd LP: 208 (ref. 15–40)	55.1 mg/dL (ref. 15–45)	n.r.	69.4 mg/dL (ref. 15–40)
CSF/serum Ig-indices	IgG normal (IgA and IgM n.r.)	n.r.	n.r.	n.r.	n.r.	IgA and IgM elevated IgG normal
cMRI	normal	subtle leptomeningeal enhancement	normal	intracranial hypertension	normal	subtle leptomeningeal enhancement
Immunusuppressive treatment	methylprednisolone	no	dexamethasone	colchicine, prednisone, (after 1.5 months AZA)	methylprednisolone	no
Recovery	complete	complete	complete	incomplete, persistent intracranial hypertension, retinitis after 1.5 months	complete	complete

n.r. = not reported; AZA = azathioprine; ^#^ = onset of meningitis-related symptoms.

## Data Availability

Datasets including biochemical, micribiological and virological results are available on request from the corresponding author.
